# Identifying neural correlates of cognitive workload in high-performance motorsport simulation: an integrated EEG and telemetry analysis of driver performance

**DOI:** 10.3389/fnrgo.2026.1765659

**Published:** 2026-03-11

**Authors:** Wasinee Terapaptommakol, Yuil Tripathee, Danai Phaoharuhansa, Tipporn Laohakangvalvit

**Affiliations:** 1 Graduate School of Engineering and Science, Shibaura Institute of Technology, Tokyo, Japan; 2 Department of Computer Engineering, King Mongkut's University of Technology Thonburi, Bangkok, Thailand; 3 Department of Mechanical Engineering, King Mongkut's University of Technology Thonburi, Bangkok, Thailand; 4 College of Engineering, Shibaura Institute of Technology, Tokyo, Japan

**Keywords:** cognitive workload profile, electroencephalography (EEG), F1 motorsport, mental fatigue, neuroergonomics

## Abstract

High-performance motorsport requires precise cognitive regulation and rapid decision-making under extreme dynamic conditions, yet traditional vehicle telemetry alone cannot reveal the psychophysiological mechanisms that influence driving performance. This study presents an integrated neuroengineering framework combining electroencephalography (EEG), and vehicle telemetry to identify objective neural markers of cognitive workload, emotional valence, and mental fatigue in a high-fidelity Formula 1 simulation. 15 participants drove on the Silverstone Circuit in the simulation platform, during which physiological data were continuously recorded and synchronized. Performance tiers were classified using k-means clustering on lap times and trajectory consistency, followed by EEG-based analysis of workload and fatigue indices. Results showed that high-performing drivers exhibited efficient workload modulation, higher alertness, and reduced fatigue compared to lower-performing drivers. A track-specific “cognitive workload profile” was also identified, revealing that technically demanding corners induced higher neural workload, whereas moderate turns corresponded to transient engagement peaks. The findings demonstrate that integrating EEG with telemetry enables objective, data-driven assessment of driver cognitive states and provides a foundation for predictive modeling, driver performance optimization, and advanced simulation-based training systems in high-performance vehicle engineering.

## 1 Introduction

High-performance driving, particularly in motorsports like Formula 1, represents a pinnacle of human-machine integration where fractions of a second determine success. A critical challenge for F1 teams is the identification and development of potential drivers, an incredibly high-stakes process involving multi-million-dollar investments in junior programs. Traditionally, driver selection relies on a combination of past race results and performance in simulators, focusing on objective vehicle telemetry (Milliken and Milliken, 1994). This method provides a wealth of data on the vehicle's state—such as speed, G-forces, and lap times. While this approach is powerful, its primary limitation is that it treats the driver as a simple input to the system. Telemetry can tell us what happened (e.g., the car braked late), but it cannot explain the core human factors—why the driver braked late, or whether they have the cognitive and emotional resilience to perform consistently under pressure (Werner et al., 2024).

Psychological factors such as cognitive load, emotional regulation under stress, and the ability to enter a state of intense focus or “flow” are understood to be decisive in these high-stakes environments (Hancock and Desmond, 2000; Melnikoff et al., 2022). Existing human factor assessments have historically relied on subjective methods, such as post-race interviews and questionnaires. While valuable for capturing perceived experience, these methods are limited by recall bias and a lack of real-time granularity, making them insufficient for the rigorous demands of talent identification (Holland et al., 2025). Furthermore, while peripheral physiological measures have been used to track stress, they often fail to differentiate between mental workload and the physical exertion inherent in racing, and they lack the temporal resolution needed to map cognitive shifts to specific track geometries.

The purpose of this study is to address these gaps through three primary objectives: first, to develop an integrated neuroengineering framework that synchronizes vehicle telemetry with EEG to objectively quantify real-time mental states and create a “cognitive workload profile” linking specific neural signatures to high-potential performance outcomes; second, to analyze driver behavior across technically demanding and high-speed pathways, clarifying how top-tier competitors adapt their neural responses to distinct circuit challenges; and finally, to provide a data-driven foundation for predictive modeling and advanced driver training systems.

## 2 Literature review

The traditional paradigm for analyzing motorsport performance has been overwhelmingly vehicle-centric. Foundational work in this area, exemplified by Milliken and Milliken (1994), focuses on vehicle dynamics, using telemetry to model and optimize the car's behavior. This approach provides indispensable, objective data on lap times, speed, and trajectory. However, it inherently treats the driver as a “black box,” an input component whose internal processes are inferred rather than measured. While telemetry can detail what a car did on the track, it offers no insight into the driver's cognitive or emotional state, which is the ultimate source of the vehicle's control (Lappi, 2018).

A paradigm shift began with the formalization of research on human factors, which posits that operator state is a critical variable in performance, particularly in high-stakes environments. The work of Hancock and Desmond (2000) was pivotal in establishing that factors such as stress, cognitive workload, and mental fatigue are not secondary considerations but primary determinants of success and failure. This recognition highlighted the need for methods that could objectively quantify these internal states in real-time, moving beyond subjective post-session reports, which are prone to recall bias.

To directly measure the operator's internal state, researchers turned to psychophysiology, beginning with measures of the autonomic nervous system. A prominent line of research involves using electrocardiography (ECG) to monitor stress and arousal during operational tasks. For example, Healey and Picard (2005) successfully used physiological sensors, including ECG, to detect stress in real-world driving conditions. Such methods provide a valuable, objective window into general physiological arousal. However, their primary limitation is a lack of specificity; it is difficult to differentiate positive emotional states (excitement) from negative ones (anxiety) or to separate mental workload from the physiological effects of physical exertion, a significant confound in motorsport (Brown et al., 2019).

To gain more specific insights into cognitive and emotional processes, the field of neuroergonomics has increasingly adopted electroencephalography (EEG). Unlike peripheral measures, EEG directly records cortical neural activity, offering unparalleled temporal resolution for tracking mental states as they unfold (Borghini et al., 2014). The foundation of EEG analysis lies in decomposing the signal into distinct frequency bands, each associated with different brain states. As summarized by Teplan (2002), these include Delta (1–4 Hz) associated with deep sleep, Theta (4–8 Hz) with drowsiness and memory consolidation, Alpha (8–12 Hz) with relaxed wakefulness, and Beta (12–30 Hz) with active thinking, concentration, and alertness. By analyzing the relative power and interplay of these bands, it is possible to derive validated indices that quantify specific, task-relevant psychophysiological states.

**Figure 1:**
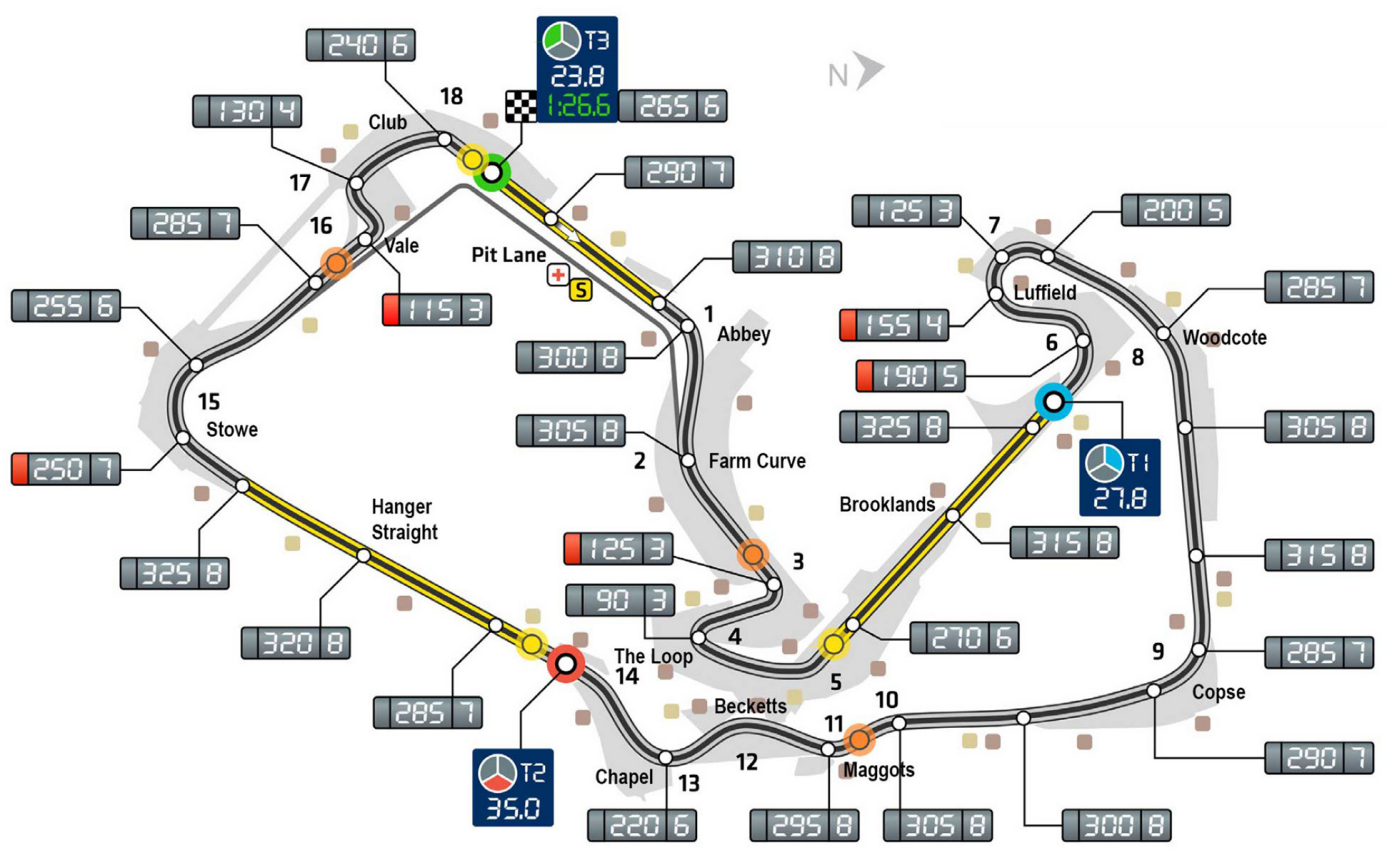
Map of the Silverstone Circuit. This schematic of the Grand Prix layout is annotated with performance data, detailing the numbered turns, key metrics such as speed (km/h), and sector times for a reference lap Silverstone Circuit map. Source: F1-Fansite.com, https://www.f1-fansite.com/f1-circuits/silverstone-circuit/ (Accessed June 2, 2025). Used with permission.

To construct a comprehensive psychophysiological profile of a driver, this study employs a carefully selected suite of eight validated EEG indices. These were selected to provide a multi-faceted view of a driver's mental state, from foundational consciousness to nuanced emotional responses (Marcantoni et al., 2023; Li et al., 2012; Zhang and Eskandarian, 2021). The core of this profile is built on tracking the critical balance between cognitive effort, alertness, and fatigue. The Workload Index (Pope et al., 1995) quantifies the mental resources allocated to driving, while the Alertness Index measures the active concentration required for rapid reactions (Meteier et al., 2021). Monitoring the Mental Fatigue Index is crucial, as its rise is a classic neural signature of declining focus and increased error likelihood (Ahn et al., 2016; Lal and Craig, 2001). Foundational to these performance metrics are indices that confirm the driver's basic operational states: the Consciousness Index provides a robust measure of wakefulness, while the Arousal/Vigilance and Relaxation/Drowsiness indices work in opposition to track the driver's position on the continuum from highly vigilant to dangerously drowsy. Beyond these cognitive states, we incorporate more nuanced measures of engagement and emotion. The Task Engagement Index offers insight into the quality of cognitive effort, helping to differentiate between strained concentration and the efficient, automatic state of “flow” (Dehais et al., 2020). Finally, Frontal Alpha Asymmetry is included to capture the driver's motivational and emotional state (Harmon-Jones et al., 2010; Di Flumeri et al., 2018), assessing the balance between approach-driven confidence and withdrawal-related stress that can fundamentally alter decision-making on the track. By simultaneously tracking these eight indices, we can move beyond a single metric to create a rich, dynamic “cognitive workload profile” of the driver, linking their real-time brain activity to specific on-track behaviors and overall performance. The key indices are summarized as in Table 1. The existing literature confirms that EEG is a powerful tool for measuring isolated cognitive and emotional states like workload, fatigue, and emotional valence in controlled and operational settings. However, a significant gap exists in applying a comprehensive suite of these validated indices within the unique, high-demand context of elite motorsport simulation. As illustrated in Figure 1, to ensure ecological validity and elicit a wide range of cognitive states, this study paired the Silverstone Circuit task environment with a commercially available, multi-channel portable EEG device. Because medical-grade EEG systems are difficult to apply in driving scenarios, this portable device was selected. Although its signal quality and number of channels are lower than those of medical-grade systems, it offers a practical trade-off between driver comfort and data-richness, allowing for the integration of multiple indices in a naturalistic simulation. This pairing of a complex task environment (Silverstone, with its well-documented balance of high-speed straights and technically demanding corners) with a practical measurement tool (a portable EEG) allows us to obtain results that are more applicable to real-world settings. Most studies focus on a single construct (e.g., fatigue) in general driving, rather than creating a holistic psychophysiological profile that can differentiate levels of expertise. The present study addresses this gap by integrating performance-based clustering with a multi-index EEG analysis to identify the distinct neural signatures that characterize high-potential drivers, providing a data-driven framework for talent identification and training.

**Table 1:** Key indices related with driving activity.

**Index name**	**Formula**	**Description and rationale**
Workload index (Pope et al., 1995)	$\frac{\beta }{\alpha +\theta }$	Measures cognitive effort and engagement. Higher value indicates greater allocation of mental resources.
Alertness index (Meteier et al., 2021)	$\frac{\beta }{\alpha }$	Measures active concentration and cortical arousal. Higher value indicates a more alert state.
Frontal alpha asymmetry (Harmon-Jones et al., 2010)	log(α_*AF*8_)−log(α_*AF*7_)	Correlates with emotional valence. Negative values suggest “approach” motivation; positive values suggest “withdrawal.”
Arousal/vigilance index (Li et al., 2012; Arns et al., 2009)	$\frac{\beta }{\theta }$	Measures physiological and mental arousal by contrasting wakefulness with drowsiness.
Relaxation/drowsiness index (Li et al., 2012; Lal and Craig, 2001)	$\frac{\theta }{\alpha }$	Detects transitions into drowsy or deeply relaxed states. High value can indicate a lapse in concentration.
Consciousness index (Teplan, 2002; Li et al., 2012)	$\frac{\alpha +\beta }{\delta }$	A robust measure of the overall state of wakefulness, contrasting waking brainwaves with sleep waves.
Task engagement index (Li et al., 2012; Pope et al., 1995)	$\frac{\theta +\alpha }{\alpha +\beta }$	A nuanced measure of engagement. A lower value indicates more effortful processing.
Mental fatigue index (Li et al., 2012)	$\frac{\theta }{\alpha +\beta }$	Quantifies cognitive exhaustion. A rising value is a classic indicator of declining focus.

## 3 Methods

### 3.1 Study design

A within-subjects experimental design was employed, where each participant acted as their own control (Lee et al., 2023; Katz et al., 2017). This design is particularly powerful for psychophysiological studies as it minimizes the influence of inter-subject variability in baseline neural activity. The primary independent variable was the track pathway section (i.e., “Straight,” “Turn 1,” and “Turn 2”), while the dependent variables included the suite of calculated EEG indices and the driver performance metrics (i.e., lap time, on-track percentage). To ensure that the observed effects were attributable to the driving task and not extraneous factors, several key variables were strictly controlled:

- **Experimental environment:** All sessions were conducted in a quiet room with sufficient lighting and a normal room temperature of 25°C to avoid outside distractions and standardize the testing atmosphere.
- **Driving task:** Task parameters were held constant for all participants. Each individual drove the same vehicle model on the Silverstone Circuit. The session consisted of a practice period lasting 30 min to 1 h (depending on participant confidence) followed by a 12-lap (approx. 20-min) experimental session.
- **Equipment:** All hardware and software were identical for every participant, including the simulator software, force-feedback steering wheel, EEG headset, and ECG sensor, thereby eliminating equipment-based variability from the data.

**Figure 2:**
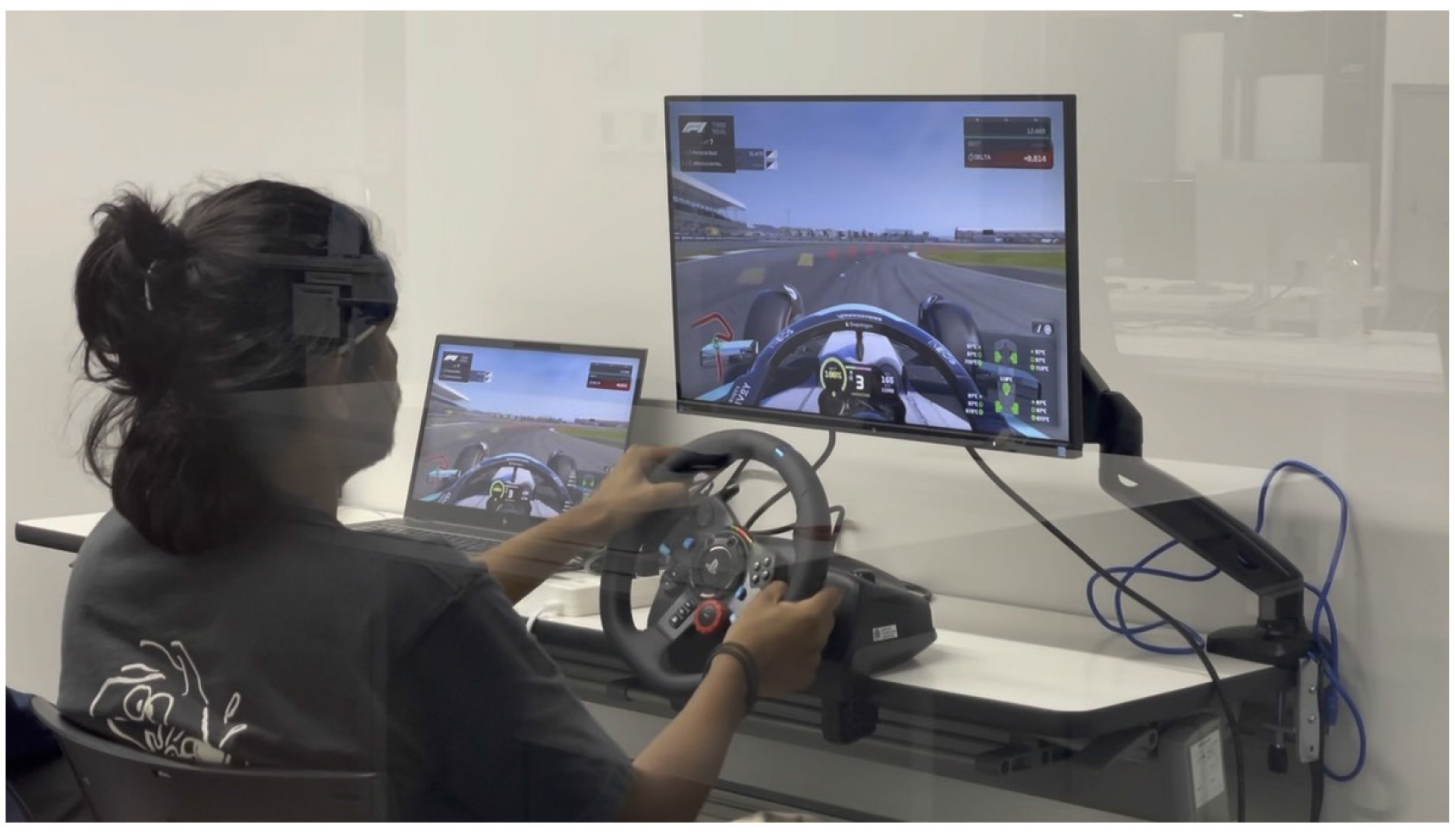
Photograph of the experiment scene. A participant equipped with a Muse S wearable EEG headband operates a high-fidelity F1 racing simulator featuring a Logitech G29 force-feedback steering system and pedals on the laser-scanned Silverstone Circuit within a controlled laboratory environment.

Figure 2 presents the experimental setup, showing the room arrangement with the device and attached sensors.

### 3.2 Participants

A total of fifteen healthy adult participants (12 males, 3 females) were recruited for this study. The participants had a mean age of 21.84 years (*SD* = 2.3). Prior to the experiment, all participants signed an informed consent form, acknowledging that their data would be collected anonymously and utilized for research purposes only. All participants possessed normal or corrected-to-normal vision and met the following criteria:

- **Inclusion criterion:** Self-reported at least 30 min of prior experience with racing simulation games to ensure a baseline level of familiarity with the task.
- **Exclusion criteria:** A history of photosensitive epilepsy; major neurological or cardiovascular disorders; or a susceptibility to severe motion sickness.

### 3.3 Materials and apparatus

Data were collected using a combination of simulation software, driver controls, and physiological sensors:

- **Simulator:** A PC-based racing F1 simulator was used with a high-fidelity, laser-scanned model of the Silverstone Circuit. A force-feedback steering wheel and pedal set were used for driver input.
- **Driver controls:** A force-feedback steering wheel and pedal set (Logitech G29) provided tactile feedback and captured driver inputs.
- **EEG system:** A Muse S wearable headset was used to continuously record EEG data from four scalp locations (AF7, AF8, TP9, TP10).
- **ECG system:** A Polar H10 wearable biosensor was used to record continuous heart rate and ECG data; however, these data were collected for synchronization purposes and future analysis and were not utilized in the current study.
- **Questionnaire:** Immediately following the experimental task, a post-task questionnaire was used to give a rating of their feelings during the entire experiment. The questions include: (1) intensity ratings for twelve discrete emotions (six positive: excited, delighted, happy, content, and relaxed, calm; six negative: tired, bored, depressed, frustrated, angry, and tense) on a 10-point scale (0 = not at all, 10 = very strongly); and (2) ratings for “driving experience,” and “car responsive feel” on an identical 0–10 scale (0 = not at all, 10 = extremely satisfying or very responsive).

### 3.4 Procedure

**Figure 3:**
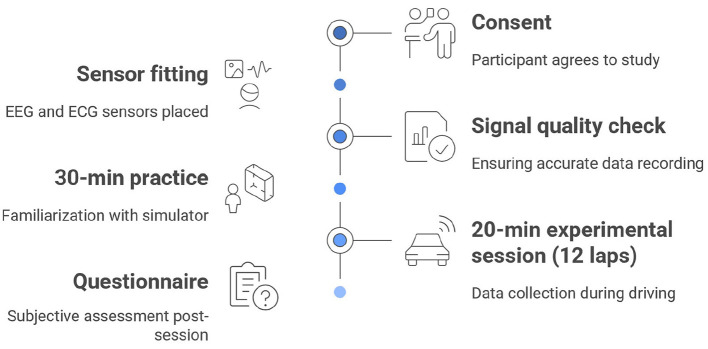
Schematic representation of the experimental procedure. Visual timeline illustrating the synchronized collection of cortical activity and vehicle telemetry data across a 12-lap session, followed by the administration of post-task subjective assessments.

The experimental procedure for each participant followed a standardized sequence, wherein each participant completed the same steps in a fixed order. This approach was implemented to ensure procedural uniformity across all sessions as shown in Figure 3.

- **Consent and sensor fitting:** After providing informed consent, the EEG and ECG sensors were fitted to the participant.
- **Signal quality check:** The quality of the physiological signals was checked to ensure stable and accurate data recording before proceeding.
- **Practice session:** Each participant completed a 30-min practice session to familiarize with the simulator, controls, and the Silverstone track layout.
- **Experimental session:** Following the practice, participants were instructed to complete 12 laps as quickly and consistently as possible. This experimental session lasted approximately 20 min. During this time, EEG, ECG, and vehicle telemetry data were continuously recorded and synchronized via a common Unix timestamp. While the ECG signal was monitored to ensure physiological stability, only the EEG and telemetry streams were processed for the performance analysis detailed in this paper.
- **Post-session questionnaire:** Immediately after the driving session, each participant completed the questionnaire to provide a subjective assessment of their emotional experience.

### 3.5 Data analysis

A multi-stage data analysis pipeline was designed to process the synchronized data streams and identify the neural signatures of high-potential drivers.

Pre-processing and Index Calculation: Raw EEG data was acquired via the Muse S headband and recorded using the Mind Monitor application. Mind Monitor adheres to the digital signal processing standards established by the original Interaxon Muse SDK (LibMuse) (Clutterbuck, 2025). At the hardware level, the signal was subjected to a notch filter (50/60 Hz) to remove environmental electrical noise and a high-pass filter to eliminate DC offset. The data was then processed using a Fast Fourier Transform (FFT) with a 256-sample sliding window to calculate Absolute Band Power for the Delta, Theta, Alpha, and Beta frequency bands. To address transient artifacts and non-physiological noise, an interpolation method was applied to the time-series data: signal segments characterized by abrupt, non-biological spikes—defined as an immediate increase and subsequent drop within a 1-second window—were identified and smoothed via linear interpolation to maintain signal continuity. This approach ensures that the subsequent spectral analysis is not biased by short-duration motion artifacts or electrode contact fluctuations.

For the purpose of index calculation in this study, spectral power analysis was concentrated on the electrodes positioned over the frontal cortex (AF7 and AF8). These spectral power values served as the basis for calculating the eight validated EEG indices, a detailed description of which is provided in the Literature Review (Table 1). These indices are primarily constructed as ratios of high-frequency to low-frequency activity to capture shifts in cognitive states. For instance, the Mental Fatigue Index was calculated as θ/(α+β), tracking the increase in low-frequency power relative to cognitive alertness. To quantify active mental effort, we utilized the Workload Index, calculated as β/(α+θ), and the Task Engagement Index, calculated as (θ+α)/(α+β). These ratios are based on the principle that increased mental demand typically leads to higher Beta activity (associated with active thinking) relative to Alpha or Theta activity (associated with relaxation or lower attention). Emotional valence was derived via Frontal Alpha Asymmetry, calculated as log(α_AF8_)−log(α_AF7_). Each index was computed for every epoch and then averaged within specific track segments to align with vehicle telemetry. To enable inter-subject comparisons of relative psychophysiological states, all final index values were normalized (z-scored) for each participant. As previously noted, although ECG data was recorded and synchronized, the present analysis focuses solely on the relationship between neural correlates (EEG) and vehicle telemetry to characterize the cognitive workload profile.

Statistical analysis: To determine the significance of the observed differences in neural signatures, a one-way Analysis of Variance (ANOVA) was performed to compare the mean values of each EEG index across the three performance groups (Potential, Normal, and No Potential). This method was selected to assess whether the variance between the performance tiers was statistically greater than the variance within the groups. Following the ANOVA, *post-hoc* pairwise comparisons (Tukey's HSD) were utilized to identify specific differences between groups where significant main effects were found. Similarly, a one-way ANOVA was conducted for the pathway-specific analysis to evaluate how neural indices fluctuated across the seven track sections, with *post-hoc* tests used to investigate significant spatial variations.

Track pathway classification: The Silverstone circuit was computationally segmented into distinct pathway types using its centerline coordinates. A “curve ratio” (curvature) was calculated for each point by analyzing the angle change relative to the distance traveled within a sliding window (8 points preceding and 8 points succeeding each point). Based on the absolute value of this curvature, each point was classified using two empirically determined thresholds:


$$\begin{array}{l} C=\frac{\text{Δ}\theta }{\text{Δ}d} \end{array}$$ (1)

**Figure 4:**
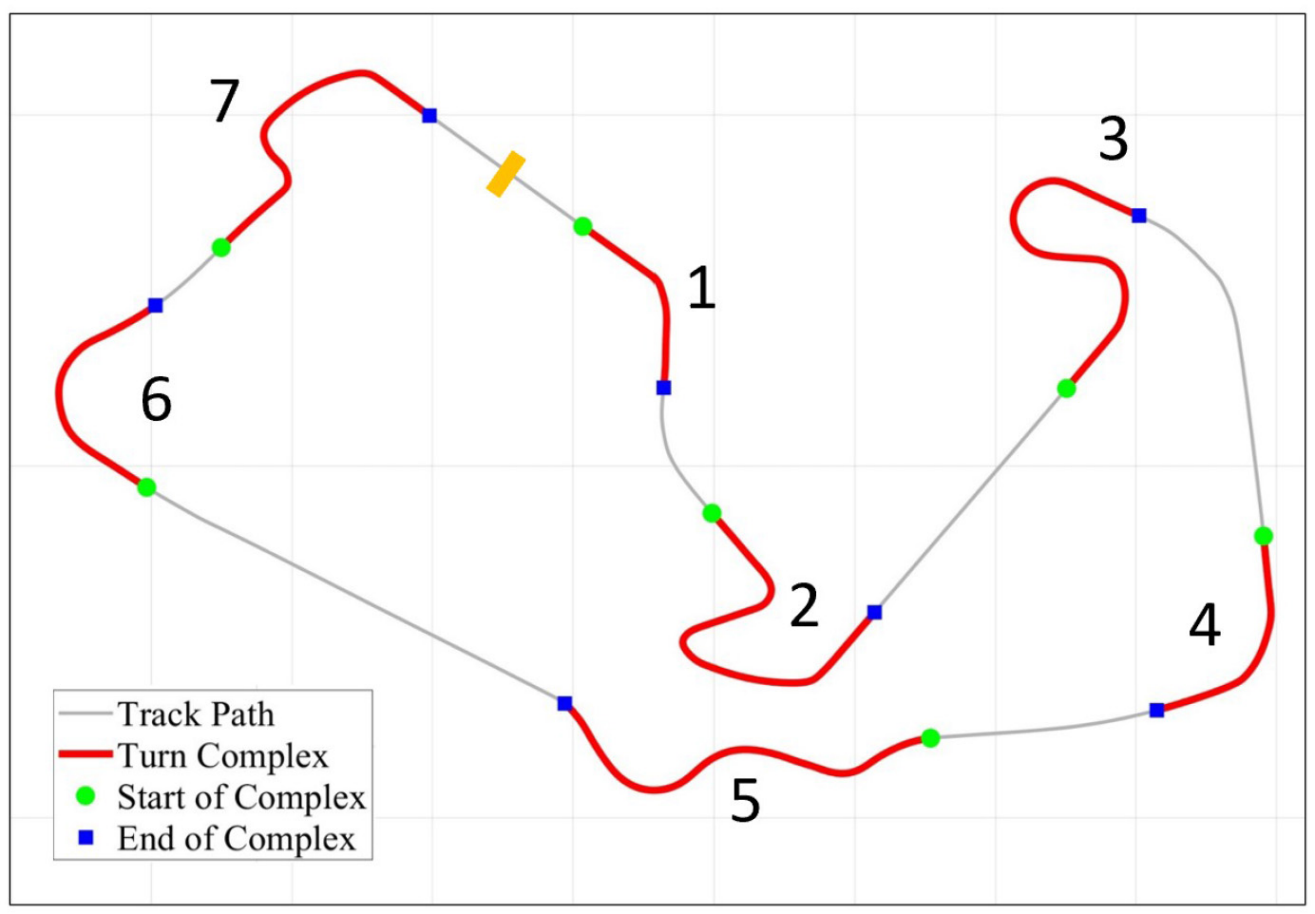
Track pathway classification of Silverstone circuit. Map of the high-fidelity racing circuit demarcating the seven specific technical segments utilized for analysis: Turn 1 (high-speed entry), Turn 2 (low-speed, narrow-radius), Turn 3 (technical S-turn), Turn 4 (high-speed, wide-radius), Turn 5 (transitional curve), Turn 6 (high-speed post-straight), and Turn 7 (low-speed final turn).

Points with a curvature less than 0.002 were labeled “Straight Way,” points with a curvature between 0.002 and 0.006 were labeled “Normal Turn,” and points with a curvature exceeding the primary threshold of 0.006 were labeled as “Shape Turn” pathways. These points were used to define seven distinct turn classifications (“Turn 1” through “Turn 7”). Based on this classification, the turns were further categorized as “Normal” (Turns 1, 4, and 6) or “Hard” (Turns 2, 3, 5, and 7). All other track sections were classified as “Straight” as shown in Figure 4.

Performance group clustering: A k-means clustering analysis was performed to categorize participants into distinct driving performance groups. The two features used for this clustering were key performance indicators: (1) total lap time and (2) percentage of time spent on the valid track pathway. The resulting clusters were labeled based on their performance characteristics: “Most Potential,” “Potential,” “Normal,” and “No Potential.”

Identification of key EEG indices: To identify which neural markers best differentiated the performance tiers, the mean values of all eight EEG indices were compared across the three performance groups (“Potential,” “Normal,” and “No Potential”). The indices that revealed the most differences between these groups were then selected for the subsequent in-depth pathway analysis.

Pathway and trend analysis: The key EEG indices identified in the previous step were analyzed across the different track pathways. This involved mapping the average value of each key index to its corresponding track section (e.g., “Straight” and “Hard Turn 2”) for each performance group. The primary goal of this analysis was to create a cognitive workload profile for the Silverstone circuit, illustrating how cognitive states like alertness, fatigue, and engagement fluctuate in response to specific driving demands. This approach allows for a direct comparison of the neural resource allocation strategies employed by different performance groups as they navigate the same course.

Subjective data analysis: Subjective scores from the post-session questionnaire were analyzed. A combined “Feel Index” was created by weighting scores for “Driving Experience” and “Car Responsive Feel.” Arousal and Valence scores from the questionnaire were also analyzed. These subjective measures were then correlated with the objective EEG indices and performance group classifications to explore the relationship between the drivers' perceived experience and their objective data.

## 4 Results

The analysis followed the multi-stage approach outlined in the Methods section, beginning with the classification of drivers into performance tiers and culminating in an analysis of their neural signatures across the racetrack. The following subsections describe the results obtained at each step of this analysis.

### 4.1 Performance group stratification and key index identification

**Figure 5:**
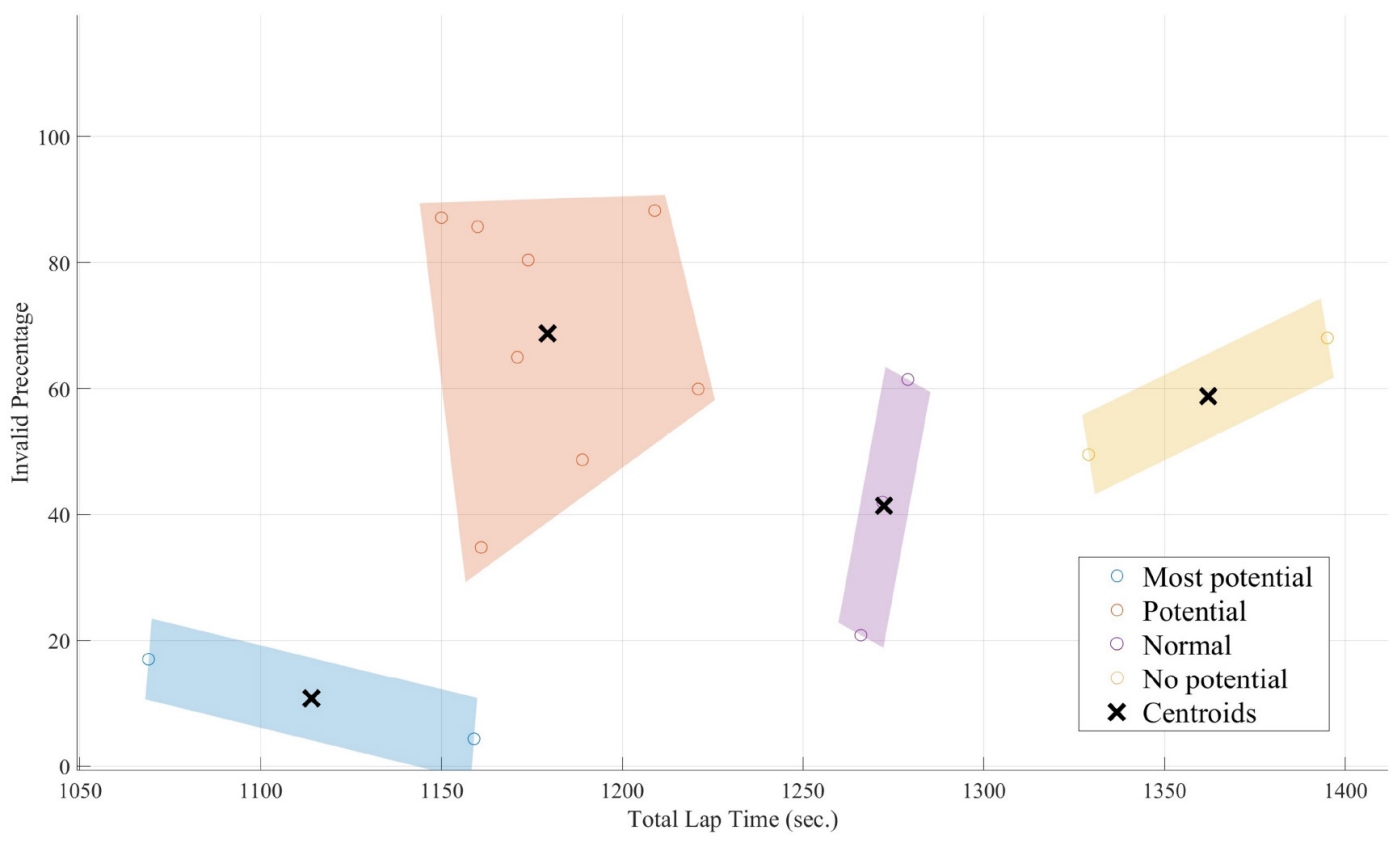
Performance group stratification via K-means clustering. Distribution of participant performance metrics and the resulting classification into Potential, Normal, and No Potential groups based on k-means clustering analysis.

The k-means clustering analysis (with k = 4) initially partitioned the 15 participants (*N* = 15) into four groups based on their driving performance characteristics. The two key metrics used for this clustering were total lap time and the invalid percentage, which represents the proportion of time a driver spent off the valid racing line or track limits. The associated number of participants classified into each group is shown in Figure 5. The performance ranges for these four initial groups were as follows:

- **Most potential (*N* = 2):** Total lap time between 1,069 seconds (17.49 minutes) to 1,159 seconds (19.19 min) with an invalid percentage of 4%–18%.
- **Potential (*N* = 8):** Lap times from 1,150 seconds (19.10 minutes) to 1,121 seconds (18.41 min) with 34%–88% invalid percentage.
- **Normal (*N* = 2):** Ranged from 1,226 seconds (20.26 min) to 1,279 seconds (21.19 min) with 20%–61% invalid percentage.
- **No potential (*N* = 3):** Ranged from 1,329 seconds (22.09 min) to 1,395 seconds (23.15 min) with 49%–68% invalid percentage.

**Figure 6:**
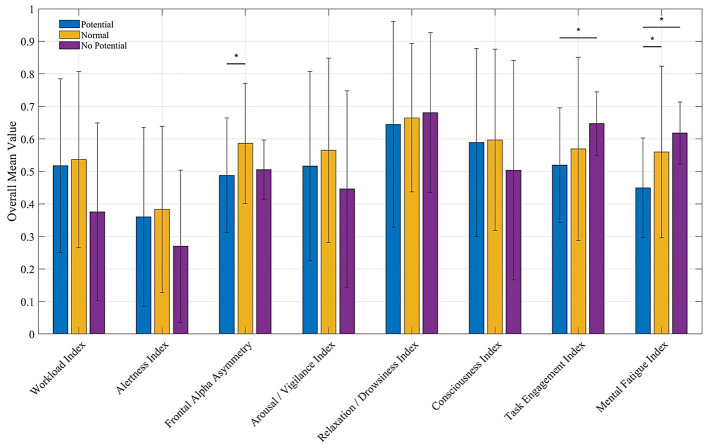
Overall average values of eight EEG indices for each of the three classification groups—Potential Group, Normal Group, and No Potential Group—across all track pathways. Error bars represent the standard deviation (SD). Significance indicators (*) above the bars represent the results of the *post-hoc* pairwise comparisons: ^*^p < 0.05.

To clarify the primary neural differentiators of driver skill, the two highest-performing groups (“Most Potential” and “Potential”) were combined into a single “Potential” group (*N* = 10). This results in three final performance tiers for comparison: “Potential,” “Normal,” and “No Potential.” This reorganization resulted in three final performance tiers for comparison: Potential, Normal, and No Potential. Statistical analysis revealed significant main effects of performance group across three primary indices: Frontal Alpha Asymmetry [$F_{\left(2,102\right)}=3.402,p=0.037,\eta^{2}=0.063$], Task Engagement [$F_{\left(2,102\right)}=10.099,p<0.001,\eta^{2}=0.165$], and Mental Fatigue [$F_{\left(2,102\right)}=18.066,p<0.001,\eta^{2}=0.262$]. *Post-hoc* Tukey's HSD tests showed that for Frontal Alpha Asymmetry, the Potential group exhibited a significantly lower index than the Normal group. For both Task Engagement and Mental Fatigue, the No Potential group exhibited significantly higher values compared to the Potential (*p* < 0.001) and Normal (*p* ≤ 0.001) tiers. The neural signatures of each group are detailed below and illustrated in Figure 6.

- **Frontal alpha asymmetry:** The “Potential” group (Avg = 0.488) showed a notably different mean value compared to the “Normal” (Avg = 0.586) and “No Potential” (Avg = 0.506) groups.
- **Task engagement index:** Lowest for the “Potential” group (Avg = 0.519), signifying a more effortful and active engagement state, compared to the “Normal” (Avg = 0.569) and “No Potential” (Avg = 0.647) groups.
- **Mental fatigue index:** Showed a clear negative correlation with performance. The “Potential” group had the lowest mean fatigue (Avg = 0.450), indicating a high resistance to cognitive exhaustion.

### 4.2 Pathway and cognitive workload analysis

To establish a neurocognitive baseline for high-performance driving, the Task Engagement Index and Mental Fatigue Index were compared across seven pathways for the Potential group (*N* = 10). These indices were selected based on their significant ability to differentiate performance tiers in the global analysis.

**Figure 7:**
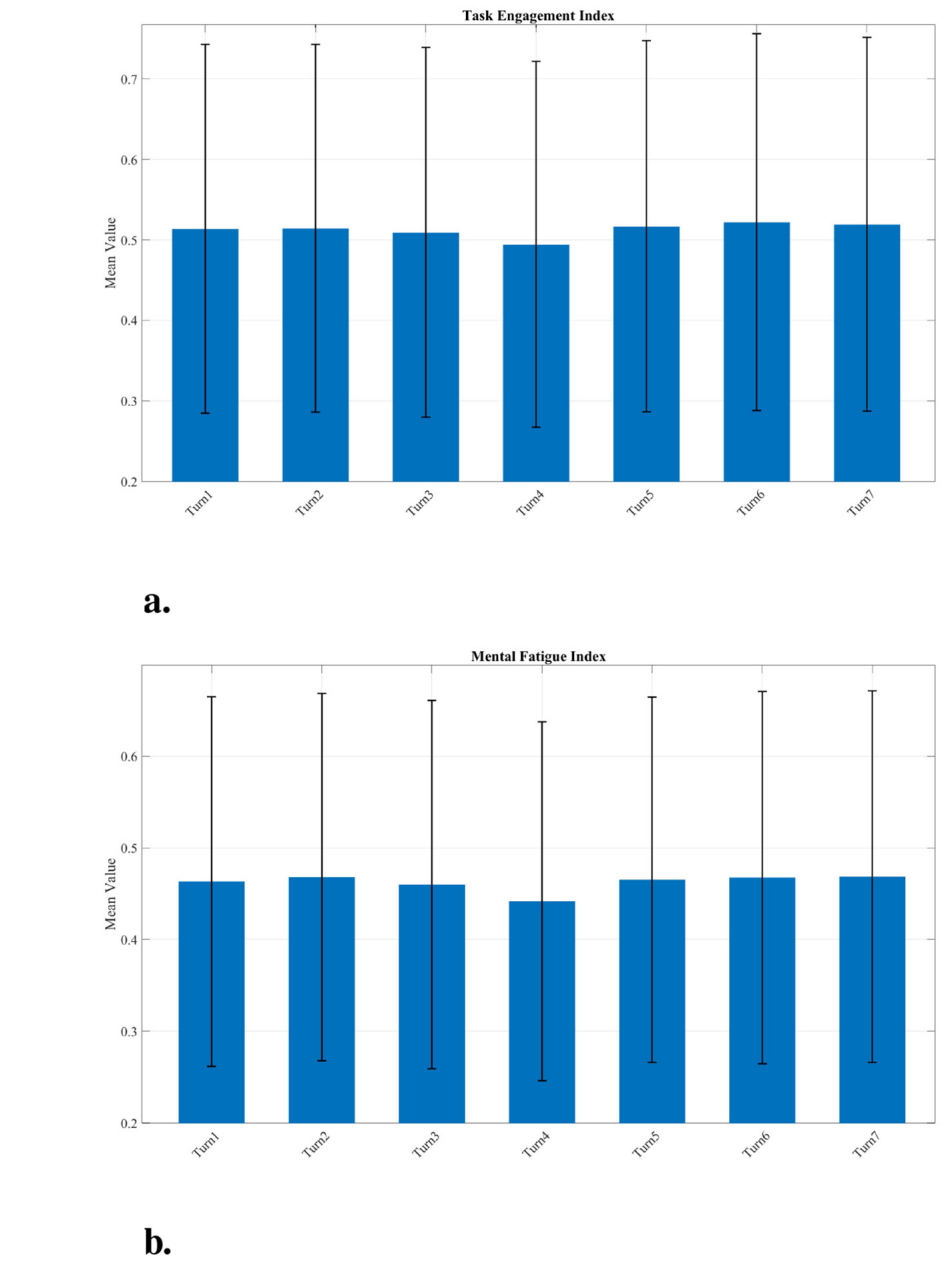
Pathway-specific variation of key EEG indices across the Silverstone circuit for the Potential Group. Mean values are presented for: **(a)** Task Engagement Index, showing descriptive peaks in mental effort (numerically lower values) during Turn 4 and Turn 3; and **(b)** Mental Fatigue Index, showing descriptive peaks in fatigue (numerically higher values) in technical sections such as Turn 7 and Turn 2. Error bars represent the standard deviation (SD). Spatial fluctuations across track sections are not statistically significant (*p* > 0.05).

Statistical comparisons across the track sections (Turns 1–7) revealed that spatial fluctuations did not reach statistical significance (*p*>0.05), with several turns exhibiting nearly identical mean values. In alignment with neuroergonomic principles, the following analysis focuses on identifying qualitative tendencies rather than absolute statistical differences. By ranking the relative mean values, we can delineate a “cognitive workload profile” of the circuit, highlighting sections where neural resources demonstrate a tendency to be more heavily taxed. The following findings detail these descriptive patterns, focusing on the two highest and two lowest-ranked sections for each index as shown in Figure 7:

- **Task engagement index:** A descriptive peak in mental effort (indicated by numerically lower index values) was observed during the navigation of Turn 4 (*Avg* = 0.494) and Turn 3 (*Avg* = 0.509). This tendency suggests that the high-speed demands of Turn 4 and the technical complexity of Turn 3 require more active cognitive recruitment from the Potential group. In contrast, index values in Turn 6 (*Avg* = 0.522) and Turn 7 (*Avg* = 0.519) were qualitatively higher, indicating a slight tendency toward a more automatic or passive processing state.
- **Mental fatigue index:** Fatigue accumulation demonstrated a descriptive tendency to peak within technically demanding, low-speed sections characterized by narrow radii, specifically Turn 7 (*Avg* = 0.469) and Turn 2 (*Avg* = 0.468). Conversely, flowing or post-recovery sections such as Turn 4 (*Avg* = 0.442) and Turn 3 (*Avg* = 0.460) recorded the lowest numerical values. This suggests that these sections function as brief recovery windows, where the transition from a long straight or the rhythmic nature of a high-speed corner allows for a “neural micro-break.” During these moments, cognitive load momentarily stabilizes, providing a transient opportunity for the brain to reset before facing the next technically demanding segment.

These neurocognitive interpretations are summarized in Table 2.

**Table 2:** Summary of pathway analysis on key EEG indices for the high-performing (potential) group.

**Index**	**Conditions eliciting peak response**	**Conditions eliciting minimum response**	**Neurocognitive interpretation**
Task engagement	Turn 3 (technical S-turn) and Turn 4 (high-speed, wide-radius) (most effortful).	Turn 6 (high-speed, post-straight) and Turn 7 (low-speed, final turn) (least effortful).	Cognitive effort descriptively peaks during high-speed navigation or technical complexity. Higher index values suggest a tendency toward automatic processing or potential overload.
Mental fatigue	Turn 7 (low-speed, final turn) and Turn 2 (low-speed, narrowest-radius).	Turn 4 (high-speed, wide-radius) and Turn 3 (post-straight, S-turn).	Fatigue accumulation tends to peak in sections requiring sustained precision. Rhythmic high-speed corners and post-straight transitions function as recovery windows for a neural micro-break and cognitive reset.

### 4.3 Fatigue trend analysis over time

**Figure 8:**
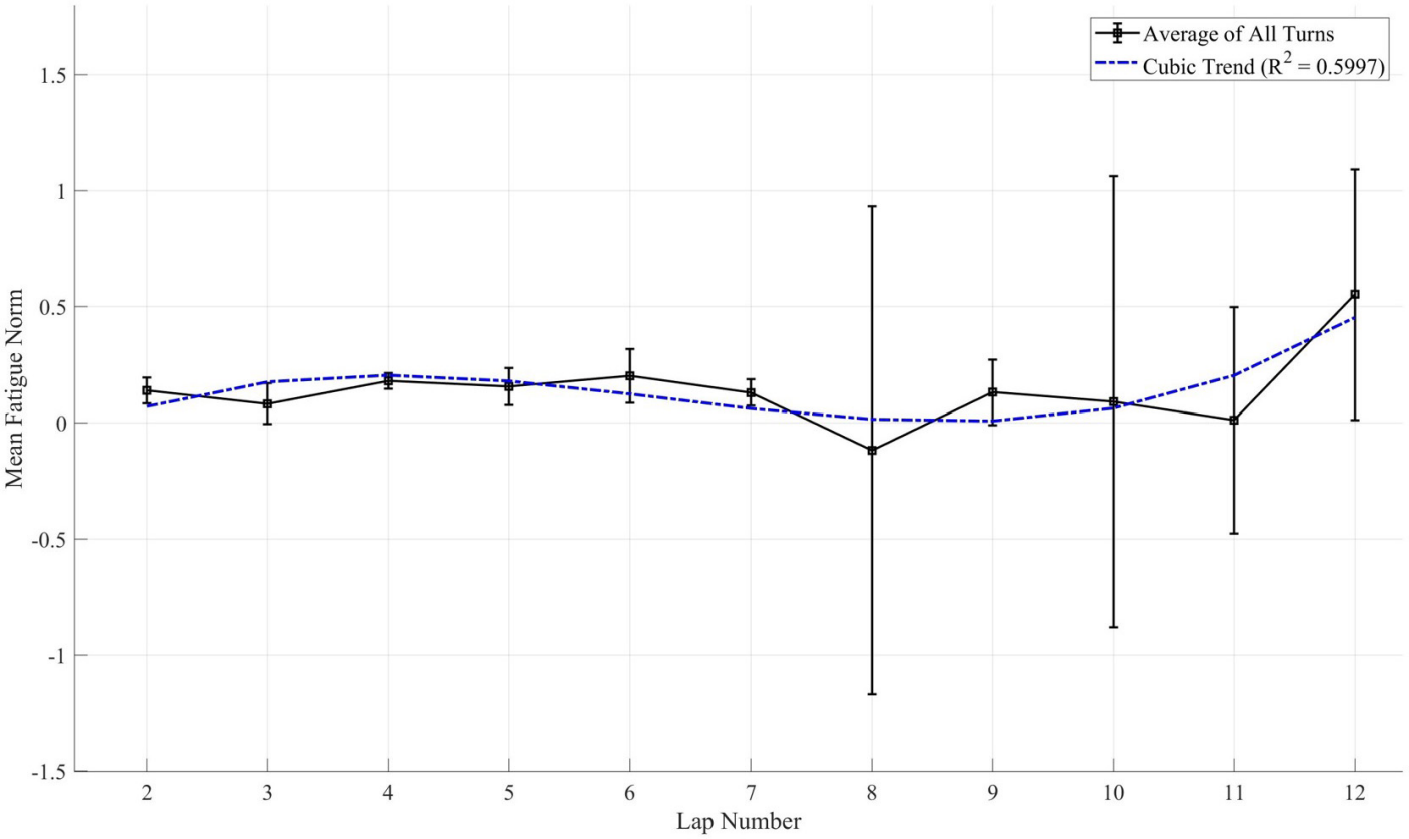
Temporal fatigue analysis across laps. Non-linear trend of the mental fatigue index across laps 2–12 for the Potential group, modeled by a 3rd-degree polynomial (*R*^2^ = 0.5997) illustrating the three-phase cognitive resource allocation process including adaptation, maintenance, and depletion.

In addition to the pathway-based (spatial) analysis, the progression of mental fatigue over the 12-lap session (temporal) was examined for the “Potential” group. A 3rd-degree polynomial trend analysis was conducted to model the change in the Mental Fatigue Index as a function of lap number (Laps 2–12). This model was found to be a moderate fit for the data (*R*^2^ = 0.5997) and approached statistical significance (*p* = 0.0783). The cubic nature of the trend (Equation 2) suggests that fatigue accumulation was non-linear, as shown in Figure 8.


$$\begin{array}{l} y=0.0039x^{3}-0.0614x^{2}+0.2623x-0.1343 \end{array}$$ (2)

## 5 Discussion

This study successfully integrated objective neurophysiological measurements with vehicle telemetry to identify the real-time neural signatures that distinguish driver performance tiers. The findings establish a rich neurocognitive profile, with statistical analysis revealing that Task Engagement, Mental Fatigue, and Frontal Alpha Asymmetry are the primary significant differentiators of driver potential. Furthermore, while spatial variations across the circuit did not reach statistical significance, the qualitative tendencies in Task Engagement and Mental Fatigue provided a descriptive “cognitive workload profile” of the Silverstone circuit. This supports the central hypothesis that high-potential driver performance is fundamentally linked to the superior modulation of neural states and the ability to exploit recovery windows during technically demanding tasks.

### 5.1 The neurocognitive profile of high-performing drivers

High-potential drivers exhibited a distinct neurocognitive phenotype characterized by three statistically significant primary indices: high fatigue resistance (Mental Fatigue Index), high effortful processing (Task Engagement Index), and a neuro-emotional state consistent with superior stress regulation (“approach” motivation; Frontal Alpha Asymmetry).

This profile provides a more nuanced understanding than traditional performance models. The Potential group's sustained effortful processing (indicated by numerically lower Task Engagement values) suggests they maintain a controlled and active cognitive state rather than relying solely on automated routines. This is fundamentally different from the signature observed in the No Potential group, which was characterized by cognitive disengagement. Low-performing drivers exhibited significantly higher Task Engagement and Mental Fatigue values compared to the Potential group. This pattern suggests that low-potential drivers become rapidly overwhelmed, leading to a state where mental effort is reduced (passive engagement) and cognitive resources are depleted, directly resulting in poor performance outcomes.

An interesting finding also emerges from the Normal group. This group exhibited the highest Frontal Alpha Asymmetry, significantly higher than the Potential group (*p* = 0.028). As noted in Table 1, higher Frontal Alpha Asymmetry is linked to “withdrawal” or stress-related emotional states. This paints a picture of a driver who is experiencing higher levels of stress and navigating the circuit less efficiently than the Potential group. The Potential group, by contrast, achieves a similar high-performance state with less perceived stress (lower Frontal Alpha Asymmetry) and superior fatigue resistance. This capacity for emotional regulation may be a critical identifier for long-term potential, as it allows drivers to maintain precision without the neuro-emotional “cost” associated with high-stress processing.

### 5.2 Heterogeneity of cognitive demand across the circuit

The analysis reveals that cognitive workload is not a monolithic construct but a dynamic state that drivers must flexibly modulate in response to changing demands. Within the Potential group, descriptive tendencies in Task Engagement and Mental Fatigue highlight how skilled individuals manage neural resource allocation across different pathway types.

As detailed in Table 2, these three indices reveal that cognitive workload is not monolithic. Descriptive peaks in effortful Task Engagement (indicated by numerically lower index values) were qualitatively allocated to the high-speed Turn 4 and the technical Turn 3. This demonstrates that active, effortful processing tends to be highest when the driver is either navigating high complexity or pushing the car toward its absolute speed limit. Conversely, qualitatively higher Task Engagement values were observed in Turn 6 (a post-straight turn) and the final Turn 7. The descriptive reduction in effortful processing in Turn 7, despite its difficulty, potentially supports the hypothesis of a cognitive overload state: after accumulating fatigue through the lap, the driver may disengage from effortful processing and shift toward a more passive, reactive mode, which can lead to errors.

Finally, Mental Fatigue accumulation was not distributed evenly across the circuit. Descriptive peaks were associated with technically demanding, low-speed turns with narrow radii (Turn 2 and Turn 7), indicating that sustained precision control under tight spatial constraints is particularly taxing for the cortex. In contrast, the flowing nature of Turn 4 and the post-straight transition of Turn 3 appear to function as “recovery windows.” These sections allow for a “neural micro-break” and a “cognitive reset,” where the rhythmic nature of the high-speed turn or the recovery from a lower-demand straight allows for a momentary stabilization of cognitive load. High-potential drivers may possess a superior capacity to utilize these brief windows to manage their finite cognitive resources, preventing the cumulative overload that characterizes lower-performing groups.

### 5.3 Temporal fatigue analysis over laps

In addition to the spatial analysis of fatigue within a lap, the analysis of mental fatigue over time revealed a second distinct pattern. The temporal analysis of fatigue across Laps 2–12 (Figure 8) revealed a complex, non-linear trend for the “Potential” group. This trend, modeled by a 3rd-degree polynomial, indicates that fatigue did not simply increase linearly. This pattern, which trended toward significance, suggests a dynamic three-phase cognitive process. The initial increase during the opening laps likely reflects the “task-loading cost” as drivers adapt from a resting state and engage sustained attentional control. This was followed by a maintenance plateau during the middle session, where the stabilization of fatigue may represent a period of optimal resource management. Finally, the rapid acceleration of fatigue during the final laps suggests a depletion of these finite cognitive resources, a “tipping point” representing the exhaustion of compensatory mechanisms that leads to a rapid decline in cognitive control and a higher probability of performance errors.

### 5.4 Limitations

While this study provides valuable insights, several limitations should be acknowledged. The sample size of 15 participants limits statistical power and generalizability. This limited number was a necessary trade-off: the experimental protocol was time-intensive for each participant, requiring both a familiarization (practice) period and a long, multi-lap testing session to acquire high-quality, detailed data. While this approach enriches the dataset for each individual, it constrains the total number of participants. Consequently, some analyses resulted in trends rather than strong statistical significance. A larger sample size would provide greater statistical power and be needed to confirm if these trending indices are definitively significant factors for differentiating driver performance.

Beyond sample size, the high-fidelity simulation environment, while offering excellent experimental control, lacks the vestibular feedback and real-world consequences (e.g., physical risk, stakes) that modulate cognitive states in actual competition. Furthermore, the 4-channel EEG system provided limited spatial resolution; higher-density systems would enable more precise mechanistic mapping. Finally, the within-subjects design may mask individual differences in cognitive strategy, as not all expert drivers may exhibit the identified neural profile.

### 5.5 Future directions

Future work should prioritize increasing the sample size, as discussed in the limitations, to provide the statistical power needed to confirm the significance of secondary markers and strengthen the generalizability of all findings. Longitudinal studies tracking drivers across seasons (i.e., following the same individuals over long-term development) would clarify whether the neural profile represents trait-like predictors of development or state-dependent expertise signatures. Intervention studies manipulating fatigue through training protocols could establish causal relationships. Cross-circuit validation would determine whether findings generalize beyond Silverstone. Integration with computational modeling and machine learning approaches applied to neural-behavioral data could identify novel patterns and yield predictive models of performance.

## 6 Conclusion

This research was motivated by the limitations of traditional, telemetry-only driver analysis, which cannot explain the critical cognitive and emotional factors that differentiate performance. The goal was to identify objective, real-time neural signatures of high-potential drivers by integrating EEG and vehicle telemetry in a high-fidelity F1 simulation. To achieve this, we grouped 15 participants into performance tiers using k-means clustering and then conducted a two-part analysis: one comparing neural profiles between performance groups and another analyzing cognitive modulation within different track pathways.

The study yielded two primary findings. First, we identified a distinct five-index neurocognitive profile for “Potential” drivers, characterized by significantly higher fatigue resistance (Mental Fatigue Index), high effortful processing (Task Engagement Index), and a low-stress emotional state (Frontal Alpha Asymmetry) when compared to other groups. This profile was fundamentally different from the “cognitively disengaged” signature of the “No Potential” group and the “inefficiently stressed” profile of the “Normal” group. Second, we established a pathway-specific “cognitive workload profile” which revealed that for skilled drivers, cognitive states are not uniform; Task Engagement and Mental Fatigue levels exhibited qualitative tendencies in response to specific on-track demands, such as the difference between high-speed corners and technical S-turns.

These findings demonstrate that integrating EEG with telemetry provides a holistic model of driver performance. This methodology offers a powerful, data-driven foundation for a new generation of talent identification protocols and advanced, cognitively-aware training systems. Future work, including expanding the sample size, will be crucial to confirm these findings and further develop this neuroengineering framework.

## Data Availability

The raw data supporting the conclusions of this article will be made available by the authors, without undue reservation.
